# White Rice Consumption and CVD Risk Factors among Iranian Population

**DOI:** 10.3329/jhpn.v31i2.16390

**Published:** 2013-06

**Authors:** Hossein Khosravi-Boroujeni, Nizal Sarrafzadegan, Noushin Mohammadifard, Firouzeh Sajjadi, Maryam Maghroun, Sedigheh Asgari, Mahmoud Rafieian-kopaei, Leila Azadbakht

**Affiliations:** ^1^Isfahan Cardiovascular Research Center, Isfahan Cardiovascular Research Institute, Isfahan University of Medical Sciences, Isfahan, Iran;; ^2^Medical Plants Research Center, Shahrekord University of Medical Sciences, Shahrekord, Iran;; ^3^Food Security Research Center, Isfahan University of Medical Sciences, Isfahan, Iran;; ^4^Department of Community Nutrition, School of Nutrition and Food Science, Isfahan University of Medical Sciences, Isfahan, Iran

**Keywords:** Cardiovascular diseases, Diet, Risk factors, White rice, Iran

## Abstract

Association between white rice intake and risk factors of cardiovascular diseases remained uncertain. Most of the previous published studies have been done in western countries with different lifestyles, and scant data are available from the Middle East region, including Iran. This cross-sectional study was conducted in the structure of Isfahan Healthy Heart Program (IHHP) to assess the association between white rice consumption and risk factors of cardiovascular diseases. In the present study, 3,006 men were included from three counties of Isfahan, Najafabad, and Arak by multistage cluster random-sampling method. Dietary intake was assessed with a 49-item food frequency questionnaire (FFQ). Laboratory assessment was done in a standardized central laboratory. Outcome variables were fasting blood glucose, serum lipid levels, and anthropometric variables. Socioeconomic and demographic data, physical activity, and body mass index (BMI) were considered covariates and were adjusted in analysis. In this study, Student's *t*-test, chi-square test, and logistic regression were used for statistical analyses. Means of BMI among those subjects who consumed white rice less than 7 times per week and people who consumed 7-14 times per week were almost similar—24.8±4.3 vs 24.5±4.7 kg/m^2^. There was no significant association between white rice consumption and risk factors of cardiovascular diseases, such as fasting blood sugar and serum lipid profiles. Although whole grain consumption has undeniable effect on preventing cardiovascular disease risk, white rice consumption was not associated with cardiovascular risks among Iranian men in the present study. Further prospective studies with a semi-quantitative FFQ or dietary record questionnaire, representing type and portion-size of rice intake as well as cooking methods and other foods consumed with rice that affect glycaemic index (GI) of rice, are required to support our finding and to illustrate the probable mechanism.

## INTRODUCTION

Cardiovascular diseases (CVDs) are the major causes of early death among men and women in developed countries (1). Increased rate of this disease has been reported from the developing countries, recently (2). CVDs are the causes of about half of deaths (43% in men and 55% in women) in European countries (2). Mortality rates due to CVDs differ with gender, age, ethnicity, socioeconomic status, and geographical region (1).

Iran, as a developing country in the Middle East, has adopted the western lifestyle both in nutritional and physical activity habits which led to a rise in the prevalence of CVDs (3). Over the last decade, CVDs have been considered the main causes of mortality in Iran (4), and the prevalence continues to rise (5). Generally, a combination of several risk factors could be considered the cause of CVDs. Smoking, obesity, high blood pressure, and cholesterol can be mentioned as the most important wellknown risk factors (6). In the development of obesity and cardiovascular diseases, dietary factors have an important role (7). In the prevention of CVDs, dietary approaches are more cost-effective, more suitable, and safer than medical approaches (8). Dietary recommendation could protect against cardiovascular disease (1,9,10). Whole grains are major components of a healthy diet in the prevention of cardiovascular diseases (11). The defending effect of whole grain-containing foods may depend on the existence or interaction of some biologically-active factors, such as dietary fibre, magnesium, vitamin E, folate, and other components (12). During the refining process, outer layer of grain containing bran and germ is separated. So, white rice that only contains the endosperm (starch) is produced, which can have adverse effects on cardiovascular disease risks and glucose metabolism (13). Fibre, minerals, vitamins, phytoestrogens, and phenolic compounds are usually found in whole grains, which are protective against diabetes and CVDs but these may be removed during the refining and milling process (14). Quality and quantity of carbohydrate which we know as glycaemic index (GI) and glycaemic load (GL) have also important role in the development of chronic diseases, particularly CVDs and diabetes (15). Foods with higher GI, as in white rice, can cause a quick postprandial increase in insulin secretion and blood glucose level and have increased risk of diabetes and cardiovascular disease in western (16) and Asian populations (17). Recently, evidence displayed that rice intake could protect against risk of mortality from cardiovascular diseases (18). So, the association between carbohydrate intake from refined grains, such as white rice and CVD risk factors still remain uncertain (18). We need more information in this regard from different regions. Interpretation of results regarding the dietary factors is usually complicated because the diets are composed of many foods containing many nutrients. Increasing consumption of refined grain in Iran could be a cause for high prevalence of CVDs. Previous investigations from Iran have shown an inverse association between consumption of whole grain and metabolic syndrome (19) as well as hypertriglyceridemic waist (20). On the other hand, most of the previously-published studies have been done in western countries with different lifestyles, and scant data are available from the Middle East region, including Iran. White rice is a major source of carbohydrate and energy intake in Iranian diet (21), and few studies have specifically focused on white rice. Therefore, the present study investigates the association between white rice consumption and CVD risk factors among Iranian population.

## MATERIALS AND METHODS

### Participants

This was a cross-sectional study done in the structure of Isfahan Healthy Heart Program (IHHP). The IHHP was a comprehensive community-based programme for prevention and control of cardiovascular diseases and promotion of healthy lifestyle. This programme was started in 1999 and concluded after 7 years in 2006 (22). The IHHP was conducted by Isfahan Cardiovascular Research Center and Isfahan Provincial Health Office together. In total, 12,514 men and women older than 19 years were selected from three cities: Isfahan, Najafabad, and Arak. Sample selection was done by multistage random cluster-sampling method. Complete information on sampling process has been presented elsewhere (22,23). This study has been done based on the available information from the initial phase of IHHP. Males with complete data on dietary and anthropometric information, plasma glucose, lipid profiles, and confounding variables were considered inclusion criteria, and history of chronic diseases and taking medication were considered exclusion criteria. As a result, 3,006 men were included in the present study. At arriving time to clinic, after full justification of the study protocol, each participant was asked to fill the consent form. The current study has received the consent of the Isfahan Cardiovascular Research Center Ethics Committee and other relevant organizations.

### Assessment of dietary intake

Dietary intakes of study participants were assessed by skilled technicians, with a 49-item food frequency questionnaire. The validity of the questionnaire was confirmed by Medical Education Development Center (22). For each item, participants were asked about the portion-sizes and frequency of consumption in the previous year. Those food items were reported as daily, weekly and monthly consumption. For analysis, all of those food items were converted to daily consumption. Each serving of white rice comprised 5 table spoonful. We also assessed quality of diet by global dietary index (GDI). The GDI represents the average of the mean of 29 questions on intake frequency in seven categories. Smaller global dietary index represents better habits (24).

### Assessment of biological factors

Participants were fasting for 12 hours before blood sampling. Collected samples were frozen at −20 °C until analysis in 72 hours at the central laboratory of Isfahan Cardiovascular Research Center. Enzymatic colorimetric method was used in measuring total cholesterol and triglyceride levels. Determination of HDL cholesterol level was done after dextran sulphate-magnesium chloride sedimentation of non-HDL cholesterol. Calculation of LDL cholesterol was done by using the Friedewald equation (25). Diabetes, high FBS level, hypertention, hypertriglyceridemia, hyperlipidemia, hypercholesterolemia, high LDL level, and low HDL level were considered CVD risk factors. Having 3 or more of the following risk factors was described as metabolic syndrome: FBS ≥126 mg/dL or waist >102 cm for men and >88 cm for women or TG ≥150 mg/dL or HDL <40 mg/dL for men and <50 mg/dL for women or systolic blood pressure ≥130 mmHg and diastolic ≥85 mmHg. Diabetes mellitus was described as FBS ≥126 mg/dL or glucose 2hpp ≥200 mg/dL or using drug for diabetes. Hypercholesterolemia was described as cholesterol ≥240 mg/dL, high LDL level as LDL ≥160 mg/dL, low HDL level as HDL >40 mg/dL for men and >50 mg/dL for women, and hypertriglyceridemia was described as TG ≥200 mg/dL. Having one disorder in the above lipid profiles was described as hyperlipidemia. High FBS level was described as FBS ≥126 mg/dL (26).

### Assessment of other variables

In a face-to-face method, skilled interviewers collected socioeconomic and demographic data (on age, education, marriage, and income), medical history, information on smoking, physical activity (using Baecke questionnaire of habitual physical activity (27), and medication. Measurement of height was done with bare feet by a metal ruler, and measurement of weight was done by a calibrated scale in light clothing. Calculation of body mass index (BMI) was done as weight (kg) divided by height (m) squared. Measurement of waist-circumference was done at a level midway between the lower rib margin and iliac crest and hip-circumference at the greatest area.

### Statistical methods

We used SPSS 15 software for all statistical analyses. Student's *t*-test was used for comparing means of continuous variables between categories of white rice intake. Chi-square test was applied to compare categorical variables. Logistic regression was used for exploring the associations between risk factors of cardiovascular diseases and category of white rice consumption in different models. First, we adjusted for socioeconomic and demographic variables, such as age, sex, education, marriage, income, smoking, and physical activity, and then a last adjustment was done for BMI. In both models, consumption of ≤7 times per week was used as a reference.

## RESULTS

Components of Iranian rice are provided in Table 1. The table has been taken from the table of Iranian food component (28). We reported different kinds of white rice which might be consumed by Iranian population.

Sociodemographic characteristics of the study participants, separately by frequency of white rice consumption, are shown in Table 2. Married people ate rice significantly less than single people. People who had higher education in terms of years of schooling consumed rice more than people with lower education. Based on our data, younger people have eaten significantly less than the elders. Eating white rice was not significantly different among those at different levels of income.

When we assessed risk factors of cardiovascular diseases among the study participants by frequency of white rice consumption per week (Table 3), we found no significant differences for glucose, lipid profiles, and inflammatory and CBC factors. We couldn't find any significant difference between BMI and waist-circumference among those at different categories of white rice intake.

Mean energy intake and global dietary index in two groups were not different (Table 4) in our study. We also couldn't see any difference for dairy, liquid oil, fruit, and vegetables in two groups but mean consumption of rice, grain, meat, sweet drink, and solid oil was significantly different, and people in 7-14 times intake group ate these items more than people in <7 times intake group.

Multivariate associations between risk factors of cardiovascular diseases and frequency of eating white rice are indicated in Table 5. We failed to find any significant association between these risk factors and frequency of white rice consumption, neither in crude model nor in adjusted models.

**Table 1.** Components of Iranian rice in 100 gramme^1^

Rice category	Energy (kcal)	Water (g)	Protein (g)	Carbohydrate (g)	Fibre (g)	Starch (g)	Total fat (g)	Thiamin (mg)	Riboflavin (mg)	Niacin (mg)	Sodium (mg)	Iron (mg)	Zn (mg)
White rice, easy cooked, boiled	138	68	2.6	30.9	1	30.9	1.3	0.01	0	0.9	1	0.2	0.7
White rice, easy cooked, raw	383	11.4	7.31	85.8	2.7	85.8	3.6	0.41	0.02	4.2	4	0.5	1.8
White rice, basmati, raw	359	10.5	7.41	79.8		79/9	0.5					1.3	
White rice, flaked, raw	346	12.6	6.6	77.5			1.2	0.21	0.05	4		8	
White rice, polished, boiled	123	69.8	2.2	29.6	.8	29.6	0.3	0.01	0.01	0.3	2	0.2	0.5
White rice, polished, raw	361	11.7	6.5	86.8	2.2	86.8	1	0.08	0.02	1.5	6	0.5	1.3
White rice, glutinous, boiled	65	83	1.7	14.7			0.3	0.02	0	0.3	1	0.2	0.4
White rice, glutinous, raw	359	13.9	8.4	74.8			1.6	0.16	0.06	2.4	3	1.2	2.2
White rice, parboiled	364	12.4	6.7	79.3	2.2	79.3	1	0.2	0.08	2.6	2	1.2	2

^1^Information in this table has been taken from the table of Iranian food component 28

## DISCUSSION

In this cross-sectional study on over 3,000 men, we failed to find any association between white rice consumption and risk factors of cardiovascular diseases. In Asian countries, like Iran, rice is a staple food. Higher consumption of white rice in these countries has been associated with higher risk of diabetes, metabolic syndrome, or CVDs (13,17,29). Whole grains, like brown rice, are rich in fibre and some plant components that are responsible for their positive effect on cardiovascular and metabolic function. During the refining process, the biologically-active factors, bran, and germ are removed, and the carbohydrate-rich endosperm section is usually retained (30).

Refined grains are typically rich in energy and poor in nutrient content, which may increase the risk of chronic diseases (31). Findings on the association between refined grain consumption and risk of chronic diseases are inconsistent (32). Studies on the associations between refined grain intake and blood pressure or risk of CVDs are less reliable than those for whole grain intake (33,34).

The results of the current study were in line with some previous studies that could not find positive association between white rice or refined grain consumption and CVD risk factors. In a study among Italians, rice intake was not associated with myocardial infarction (35). In another study on Indians, rice intake, in comparison with other sources of carbohydrate, had lower potential to increase postprandial glycaemia and TG levels (36). McKeown *et al*. reported no association between refined grain intake and metabolic risk factors (11). Other studies also had no evidence for effect of refined grain on heart disease (37-39), type 2 diabetes (16,40), mortality due to CVDs (41), and risk of hypertension (42). Epidemiologic studies also reported that refined grain intake is not associated with risk of type 2 diabetes (40).

Conversely, in some studies, refined grain intake was positively associated with the higher risk of metabolic diseases (19). According to some reports, refined grain intake was associated with higher fasting glucose level (41), higher hyperinsulinemia (43), risk of type II diabetes (16,44,45), stroke (46), and hypertriglyceridemia (20). Cross-sectional studies have reported a direct association between refined grain intake and prevalence of metabolic syndrome (19,41,47). White rice intake was adversely associated with mortality from coronary heart disease (CHD), heart failure, and CVD risk in men (18,48). Two cohort studies also reported an inverse association between carbohydrate intake, mostly rice, and CHD risk factors among men (49,50).

**Table 2.** Characteristics of study participants by frequency of white rice consumption per week^1^

Variable	White rice intake		p value
	<7 times per week	7-14 times per week	
Physical activity (mets.h/day)	1102.2±537.6	1063.6±545.0	0.40
Married (%)	79	72	<0.05
Education (%)			
0-<6 years	38	28	<0.05
6-12 years	48	53	
>12 years	14	19	
Age (years)	39.0±15.2	34.5±13.2	<0.001
Age (%)			
19-<25 years	92	8	<0.001
25-<35 years	93	7	
35-<45 years	95	5	
45-<55 years	96	4	
55-65 years	96	4	
>65 years	98	2	
Income (%)			
US$ <100 per month	94	6	0.22
US$ 100-300 per month	93	7	
US$ ≥300 per month	100	0	

^1^Data are means±standard deviation unless indicated; Mets.h/day=Metabolic equivalent.hours per day

**Table 3.** Risk factors of cardiovascular diseases among study participants by frequency of white rice consumption per week^1^

	White rice intake		p value
	<7 times per week	7-14 times per week	
Fasting blood sugar (mg/dL)	83.2±29.3	82.0±21.1	0.60
Glucose (2hpp) (mg/dL)	97.6±50.6	95.9±44.6	0.66
Cholesterol (mg/dL)	197.9±49.1	197.7±52.7	0.94
Triglycerides (mg/dL)	188.3±126.6	177.4±116.2	0.28
HDL (mg/dL)	45.5±9.9	45.4±10.0	0.85
LDL (mg/dL)	116.2±40.0	118.1±46.3	0.57
Apolipoprotein A (mg/dL)	142.6±31.2	142.0±36.7	0.67
Apolipoprotein B (mg/dL)	113.2±31.7	107.6±22.9	0.34
CRP-quantitative (mg/dL)	3.1±1.2	3.0±1.0	0.94
RBC (x106/µL)	5.3±0.5	5.2±0.4	0.35
Haemoglobin (g/dL)	15.2±1.3	15.1±1.2	0.38
Haematocrit (%)	45.6±3.5	45.3±3.3	0.30
Body mass index (kg/m^2^)	24.8±4.3	24.5±4.7	0.30
Waist-circumference (cm)	90.6±89.5	89.5±11.5	0.24
Waist-to-hip ratio	0.9±0.1	0.9±0.1	0.27

^1^Data are means±standard deviation

**Table 4.** Energy and dietary intake of study participants by frequency of white rice consumption per week^1^

	White rice intake		p value
	<7 times per week	7-14 times per week	
Energy (kcal/day)	1839.7±776.7	2106.5±634.5	0.19
Global dietary index	1.1±0.3	1.1±0.3	0.20
Solid oil^2^	6.43±4.0	7.8±5.0	<0.001
Liquid oil	2.6±3.5	2.8±3.7	0.34
Grain	2.8±2.0	3.6±3.3	<0.05
Fruit	6.2±3.5	6.5±4.3	0.29
Vegetables	5.4±3.1	5.8±4.2	0.16
Meat	5.8±3.0	7.1±3.8	<0.001
Dairy product	1.5±2.6	1.7±3.0	0.36
Sweet drink	1.9±2.2	2.6±3.0	<0.05
Rice and bread	22.4±5.9	30.5±6.1	<0.001

^1^Data are means±standard deviation;

^2^Dietary intakes are times per week

As mentioned, results about the association of refined grains and risk factors of chronic diseases are contradictory (32). This difference might be explained by the diversity in glycaemic indices of different refined grains as the GI of different rice categories among the Vietnamese is from 86 to 109 but, based on the results of the systematic review, the mean GI for white rice was 64 and for brown rice was 55 (51). High-GI diet generates a high postprandial blood glucose level which produce a high insulin demand and metabolic risk for long time (52,53). A high GI and also high amount of carbohydrate diet (GL) have been associated with higher risk of metabolic syndrome (47). In a cohort study on the Chinese, intake of food high in GI or GL, particularly rice, was positively associated with risk of type 2 diabetes (17). Foods containing refined grains are major suppliers of high-GL foods, and several studies revealed that GL is positively associated with lower glycaemic control or metabolic risk (54,55).

Although nobody can deny the result of previous studies on the association between white rice intake and risk of metabolic syndrome and the beneficial effect of whole grain, we did not find any relationship between white rice and CVD risk factors. One possible reason for not finding positive association between white rice and CVD risk factors in our study could be the GI in Iranian rice as well as cooking method. Previous studies on Iranian rice determined some kinds of low-GI rice. Darabi *et al*. (56) reported that GI of rice Binam was 44±9 and, in another study (57), GI of Sorna Pearl rice was reported to be 52.2±5.1. They assumed that the low GI of these kinds of rice could be because of different cooking methods and amylase content in Iranian rice (56). Although white rice, in comparison with brown rice, has no biologically-active agents, lower GI in Iranian rice could be a compensation factor that diminishes the negative effect of this refined grain.

### Limitations

There are several limitations in our study that should be considered. First, our study had a cross-sectional design. Thus, our findings do not allow us to make conclusion about the causality. Future prospective studies may give stronger documentation. On the other hand, we used FFQ for the dietary assessment as previous studies explained; FFQ would lead to under-estimation of refined grain intakes (58). The GI and GL of the white rice were not measured in the present study.

### Conclusions

Grains as a main source of carbohydrate have important role in the diet of different populations, especially among the Asians. Moreover, importance of biologically-active and other components in whole grains is not deniable. There was no significant association between white rice intake and CVD risk factors among Iranian men. Further prospective studies with a semi-quantitative FFQ or dietary record questionnaire, representing type and portion-size of rice intake as well as cooking methods and other foods consumed with rice that affect GI of rice are required to support our finding and to illustrate the probable mechanism.

**Table 5.** Multivariate adjusted odds ratios for risk factors of cardiovascular diseases by frequency of white rice consumption per week

	White rice intake		p value
	<7 times per week	7-14 times per week	
Metabolic syndrome^3^			
Crude	1.00	0.85 (0.53-1.35)	0.45
Model 1^1^	1.00	1.22 (0.74-2.02)	0.42
Model 2^2^	1.00	1.25 (0.72-2.18)	0.42
Diabetes Mellitus^4^			
Crude	1.00	0.68 (0.32-1.48)	0.33
Model 1	1.00	1.07 (0.48-2.42)	0.87
Model 2	1.00	1.06 (0.47-2.43)	0.88
Hypercholesterolemia^5^			
Crude	1.00	1.12 (0.76-1.66)	0.56
Model 1	1.00	1.29 (0.86-1.94)	0.23
Model 2	1.00	1.19 (0.77-1.84)	0.44
High LDL level^6^			
Crude	1.00	0.87 (0.53-1.42)	0.57
Model 1	1.00	1.03 (0.61-1.73)	0.91
Model 2	1.00	0.90 (0.52-1.56)	0.70
Low HDL level^7^			
Crude	1.00	1.17 (0.84-1.63)	0.35
Model 1	1.00	1.09 (0.77-1.56)	0.60
Model 2	1.00	1.10 (0.78-1.57)	0.58
Hypertriglyceridemia^8^			
Crude	1.00	0.75 (0.53-1.07)	0.11
Model 1	1.00	0.80 (0.56-1.16)	0.24
Model 2	1.00	0.78 (0.52-1.16)	0.21
Hyperlipidemia^9^			
Crude	1.00	0.96 (0.70-1.30)	0.78
Model 1	1.00	0.98 (0.71-1.36)	0.90
Model 2	1.00	0.96 (0.68-1.34)	0.79
High FBS level^10^			
Crude	1.00	0.56 (0.20-1.53)	0.26
Model 1	1.00	0.84 (0.30-2.38)	0.74
Model 2	1.00	0.85 (0.30-2.44)	0.77

^1^Model 1: Adjusted for sociodemographic variables;

^2^Model 2: Adjusted for sociodemographic variables and BMI;

^3^Having 3 or more factors: FBS >126 mg/dL or waist-circumference >102 cm for men and >85 cm for women or TG >150 mg/dL or HLD <40 mg/dL for men and <50 mg/dL for women or systolic blood pressure >130 mmHg and diastolic > 85 mmHg;

^4^FBS >126 mg/dL or glucose 2hpp >200 mg/dL or using of hypoglycaemic agents;

^5^Cholesterol >240 mg/dL;

^6^LDL >160 mg/dL;

^7^HDL >40 mg/dL for men and >50 mg/dL for women;

^8^TG >200 mg/dL;

^9^Having one disorder in the above lipid profiles;

^10^FBS >126 mg/dL

## ACKNOWLEDGEMENTS

The study used data from the programme conducted by Isfahan Cardiovascular Research Center (ICRC)—a WHO collaborating centre—in collaboration with Isfahan Provincial Health Office, both affiliated to Isfahan University of Medical Sciences (IUMS). The programme was supported by a grant (No. 31309304) of the Iranian Budget and Planning Organization as well as the Deputy for Health of the Iranian Ministry of Health, Treatment and Medical Education, and the Iranian Heart Foundation (IHF). We are thankful to the collaborating teams in ICRC, Isfahan Provincial Health Office, Najafabad Health Office, and Arak University of Medical Sciences.
